# Mosaicism for Cohen–Gibson Syndrome in a Female Individual With Overgrowth and Neurodevelopmental Delay

**DOI:** 10.1002/mgg3.70286

**Published:** 2026-10-05

**Authors:** Fei Song, Manuel Michels, Beate Betz, Walburga Vorhoff, Teresa Neuhann, Lisa Senninger, Dagmar Wieczorek

**Affiliations:** ^1^ Institute of Human Genetics, Medical Faculty and University Hospital Düsseldorf Heinrich‐Heine‐University Düsseldorf Düsseldorf Germany; ^2^ Private Practice in Pediatric Endocrinology and Diabetology Düsseldorf Germany; ^3^ MGZ—Medical Genetics Center Munich Germany

**Keywords:** Cohen–Gibson syndrome, *EED*, exome sequencing, overgrowth syndrome, somatic mosaicism

## Abstract

**Background:**

The Embryonic Ectoderm Development (*EED*) gene encodes a core component of the Polycomb Repressive Complex 2 (PRC2), which regulates chromatin structure and plays a key role in embryonic development and cell differentiation. Pathogenic variants in *EED* are associated with overgrowth–intellectual disability syndrome (Cohen–Gibson syndrome, OMIM #617561), though reported cases are rare and typically involve heterozygous germline variants. This study presents an individual with confirmed postzygotic mosaicism in *EED*, contributing to diagnostic complexity.

**Methods:**

A 12‐year‐old girl with tall stature, dysmorphic features, developmental delay, and congenital anomalies underwent clinical and genetic evaluation. Exome sequencing and targeted deep sequencing were used to identify and validate the genetic variant across multiple tissues.

**Results:**

A likely pathogenic mosaic *EED* variant c.1091G > T; p.(Trp364Leu) was identified in the patient with a 31% variant allele fraction in peripheral blood. Deep sequencing confirmed the variant and its de novo origin in the parents' samples. Mosaicism was also detected in two independently collected buccal mucosa samples (dry swab and buccal swab collection kit; 11% and 17%) and in saliva (30%), indicating somatic mosaicism in multiple tissues. DNA methylation profiling revealed a high‐confidence epigenetic signature consistent with a *PRC2* complex‐associated disorder, further supporting the likely pathogenic classification of the identified variant.

**Conclusion:**

This case broadens the molecular understanding of *EED*‐related disorders and highlights the clinical significance of mosaicism. Deep sequencing and multi‐tissue analysis are crucial for accurate diagnosis, management, and genetic counseling in overgrowth syndromes.

## Graphical abstract

Postzygotic *EED* mosaicism: c.1091G > T; p.(Trp364Leu) in a 12‐year‐old female with overgrowth–intellectual disability syndrome (Cohen–Gibson syndrome). Variant allele fractions: 31% blood, 30% saliva, 11%–17% buccal mucosa. This expands the spectrum of *EED*‐related disorders, highlighting mosaic screening for diagnosis, counselling, and future therapies. Created in part with BioRender.com.

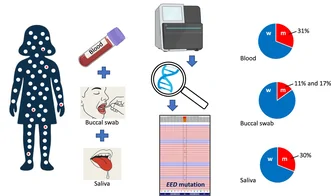

## 1 Introduction

Embryonic Ectoderm Development (*EED*), along with Enhancer of Zeste Homolog 2 (*EZH2*), Suppressor of Zeste 12 Homolog (*SUZ12*), and Retinoblastoma Binding Proteins 4 and 7 (RBBP4/7), forms the core of the Polycomb Repressive Complex 2 (PRC2) (Margueron and Reinberg 2011). This complex mediates epigenetic gene silencing through tri‐methylation of histone H3 at lysine 27 (H3K27me3), leading to chromatin compaction and transcriptional repression (Cao et al. 2002). EED plays a key role by recognizing H3K27me3 marks, which helps propagate and maintain the repressed chromatin state. This mechanism is essential for proper regulation of developmental genes and the preservation of cellular identity (Margueron et al. 2009).

Pathogenic variants in components of the Polycomb Repressive Complex 2 (PRC2) have been implicated in a spectrum of overgrowth syndromes characterized by increased stature, macrocephaly, advanced bone age, distinctive facial features, and varying degrees of intellectual disability (Cyrus, Burkardt, et al. 2019). Specifically, heterozygous pathogenic germline variants in *EZH2* are associated with Weaver syndrome (Gibson et al. 2012; Tatton‐Brown et al. 2011), OMIM #277590, characterized by tall stature, macrocephaly, advanced bone age, and distinctive facial features. Similarly, heterozygous variants in *EED* and *SUZ12*, other core components of PRC2, have been linked to overgrowth syndromes. Pathogenic variants in *EED* are associated with Cohen–Gibson syndrome (Cohen and Gibson 2016; Cohen et al. 2015), while *SUZ12* variants have been implicated in Imagawa‐Matsumoto syndrome (Imagawa et al. 2017), OMIM #618786. These conditions share phenotypic features with Weaver syndrome, including overgrowth and intellectual disability, although they are less well characterized due to their rarity (Cyrus, Cohen, et al. 2019; Sequerra Amram Cohen and Gibson 2019).

The *EED*‐related overgrowth syndrome, Cohen–Gibson syndrome, is a rare congenital disorder resulting from heterozygous pathogenic variants in the *EED* gene. Clinically, individuals with *EED*‐related overgrowth syndrome exhibit phenotypic features that overlap substantially with Weaver and Sotos syndromes, including prenatal and postnatal overgrowth, advanced bone age, macrocephaly, hypotonia, and intellectual impairment with variable severity, ranging from mild to profound (Kurotaki et al. 2002; Weaver et al. 1974; Weisswichert et al. 1981). The facial gestalt of individuals with *EED*‐related overgrowth syndrome can generally be distinguished from that of Weaver syndrome, particularly at older ages, with *EED* cases showing different features such as telecanthus, narrow palpebral fissures, ptosis, downslanted upper eyelids, and eversion of the lower lateral eyelid margins (Griffiths et al. 2019). Importantly, the phenotypic spectrum associated with disease‐causing *EED* variants is variable. In addition to neurodevelopmental delay, some individuals present with congenital heart defects, genitourinary anomalies, structural brain abnormalities, and epileptic seizures, reflecting variable expressivity and potential pleiotropy of *EED* pathogenic variants (Cohen and Gibson 2016; Cohen et al. 2015; Cooney et al. 2017).

To date, mosaicism involving pathogenic variants in the *EED* gene has not been reported in association with overgrowth syndromes. All 21 published individuals with *EED*‐related conditions, including Cohen–Gibson syndrome, are reported to carry heterozygous germline pathogenic variants, most commonly arising de novo (Cohen and Gibson 2016; Cohen et al. 2015; Cooney et al. 2017; Fu et al. 2022; Goel et al. 2024; Griffiths et al. 2019; Hetzelt et al. 2022; Imagawa et al. 2017; Kaplanis et al. 2020; Levy et al. 2022; Spellicy et al. 2019; Tatton‐Brown et al. 2017; Yu et al. 2012; Zhou et al. 2022). Variant databases such as HGMD exclusively list germline alterations, either de novo or inherited. In contrast, postzygotic mosaicism is a well‐established mechanism in other overgrowth syndromes, particularly those caused by somatic activating variants in *PIK3CA* or *AKT1* (Kurek et al. 2012; Lindhurst et al. 2011; Luks et al. 2015). The identification of a patient harboring a likely pathogenic *EED* variant in mosaic form is therefore of particular clinical and scientific interest.

In this report, we describe a female patient with clinical features consistent with an overgrowth syndrome, in whom a likely pathogenic *EED* variant was identified in mosaic form. The variant was confirmed by next‐generation sequencing in multiple tissues, supporting its postzygotic origin. DNA methylation analysis revealed an epigenetic profile matching the episignature associated with *EED*‐related disorders, reinforcing the variant's likely pathogenic classification. This finding expands the known mutational spectrum of *EED*‐associated disorders and highlights the importance of considering mosaicism in individuals with atypical or unexplained clinical presentations.

## 2 Materials and Methods

### 2.1 Ethical Compliance and Clinical Evaluation

This study was conducted in accordance with the principles of the Declaration of Helsinki. Ethical approval was obtained from the responsible ethics committee of Heinrich Heine University (Study ID: 2018094828). Written informed consent was provided by the legal guardians for genetic testing and for the publication of anonymized clinical and molecular data. Written informed consent was also obtained for the publication of clinical photographs.

### 2.2 DNA Extraction and Whole‐Exome Sequencing

Genomic DNA was extracted from peripheral blood leukocytes using the QIAamp DNA Blood Mini Kit (Qiagen). Whole‐exome sequencing (WES) was performed on a DNA sample from the patient. Exonic regions were enriched using the Twist Comprehensive Exome MT kit (target region: 36.8 Mb) and sequenced on the Illumina NextSeq 550 platform, generating paired‐end reads.

Bioinformatic analysis of the sequencing data was performed using an integrated pipeline implemented in dedicated software (Varfeed and Varvis, Limbus Biosciences, Rostock, Germany). This included raw data processing, alignment to the GRCh38/hg38 human reference genome, variant calling, and annotation of single nucleotide variants (SNVs), insertions/deletions (indels), and copy number variants (CNVs). The analysis included the entire exome, incorporating Human Phenotype Ontology (HPO) terms and a panel of genes commonly associated with overgrowth–intellectual disability syndromes, including *EZH2*, *NSD1, DNMT3A, PTEN*, *SUZ12*, among others. All were negative for rare or clinically significant variants.

### 2.3 Targeted Nextera Deep Sequencing and Mosaicism Assessment

To confirm the presence of the suspected variant in the index patient and her biological parents, and to assess potential tissue‐specific mosaicism, targeted high‐depth sequencing was performed in different tissues.

Genomic DNA from the parents was extracted from peripheral blood leukocytes using the QIAamp DNA Blood Mini Kit (Qiagen). In the patient, DNA was isolated from three additional tissue sources: two independent buccal mucosa samples and one saliva sample. The first buccal sample was collected using a dry sterile swab and processed with the Monarch Genomic DNA Purification Kit (New England Biolabs). The second buccal sample was collected using an Oracollect•DNA OCR‐100 swab (DNA Genotek) and extracted using the prepIT•L2P DNA Isolation Kit (DNA Genotek). The saliva sample was collected using an Oragene•DNA OG‐500 self‐collection kit (DNA Genotek), and DNA was isolated using the prepIT•L2P kit, following the manufacturer's protocol.

Custom primers were designed to amplify the genomic region containing the identified *EED* variant. Subsequent analysis of the PCR fragments was performed using the Illumina DNA Prep Kit (M) Tagmentation with Nextera DNA CD indices. Sequencing was carried out with the MiSeq Reagent Nano Kit v2 on the Illumina MiSeq instrument (Illumina, San Diego, USA). The sequences were aligned to the human genome reference NCBI37/hg19. Local Run Manager software, version 3.0.0 (Illumina, San Diego, CA, USA) was used for run setup and secondary analysis. The sequencing data were analyzed using the IGV software (Integrative Genomics Viewer). The method reliably detects variants with a variant allele fraction of ≥ 1%, whereas variants below this threshold may not be detected reliably.

### 2.4 Episignature

Genome‐wide DNA methylation profiling was performed using the Infinium Methylation EPIC BeadChip (Illumina Inc., San Diego, CA, USA). Genomic DNA was bisulfite‐converted using the EZ DNA Methylation Kit (Zymo Research, Irvine, CA, USA) and processed according to the manufacturer's protocol. Arrays were scanned using the iScan system, and raw intensity data (IDAT files) were generated.

Secondary analysis of the IDAT files was performed using the cloud‐based EpiSign METRIC software (EpiSign Inc., Canada), an automated analytics software for methylation‐based episignature classification. This platform compared genome‐wide DNA methylation profiles against a reference database of established episignatures. The classification algorithm integrated multiple statistical and analytical parameters, including hierarchical clustering, multidimensional scaling (MDS), and t‐distributed stochastic neighbor embedding (t‐SNE), in combination with support vector machine (SVM)‐based methylation variant pathogenicity (MVP) scoring.

## 3 Results

### 3.1 Clinical Findings

The female index patient was born at 36^+3^ weeks of gestation via primary cesarean section due to maternal edema and hypertension. The pregnancy was otherwise unremarkable and family history was uneventful. The index patient is the only affected individual in the family across three generations.

At birth, the patient exhibited macrosomia, with a weight of 4500 g (+3.76 SD), length of 55 cm (+2.43 SD), and head circumference of 38 cm (+3.05 SD). Postnatal hypotonia was noted.

Early developmental milestones were significantly delayed. Walking without support was achieved at approximately 24 months of age, and first spoken words emerged around 30 months. She is currently enrolled in the 6th grade at a school for children with intellectual disabilities. Multidisciplinary interventions were initiated early and included physiotherapy, occupational therapy, speech therapy, and early childhood developmental support. She qualifies for care level 3 under the German statutory long‐term care insurance system, indicating a substantial need for assistance with daily living activities due to health‐related impairments of independence or abilities.

At the age of 12 years, facial dysmorphic features included hypertelorism, narrow palpebral fissures, periorbital fullness, and large, posteriorly rotated ears. Additional phenotypic anomalies included genu valgum, thin calves, and slender fingers with short first and fifth metacarpals, resulting in a short thumb and fifth finger (Figure 1). Neurological examination revealed increased patellar reflexes. Cardiological evaluation identified a systolic murmur, and echocardiography confirmed valvular insufficiency (Table 1). Clinical evaluation was performed due to tall stature, global developmental delay, distinct dysmorphic features, and multiple congenital anomalies (Figure 1). Her height measured 173.5 cm (+2.97 SD), head circumference 55.5 cm (+1.64 SD), and weight 57.2 kg (+1.54 SD). Arm span was 173 cm, and leg length was 97.5 cm, indicating proportionate overgrowth. Tanner staging was B2–3 and PH1.

**FIGURE 1:**
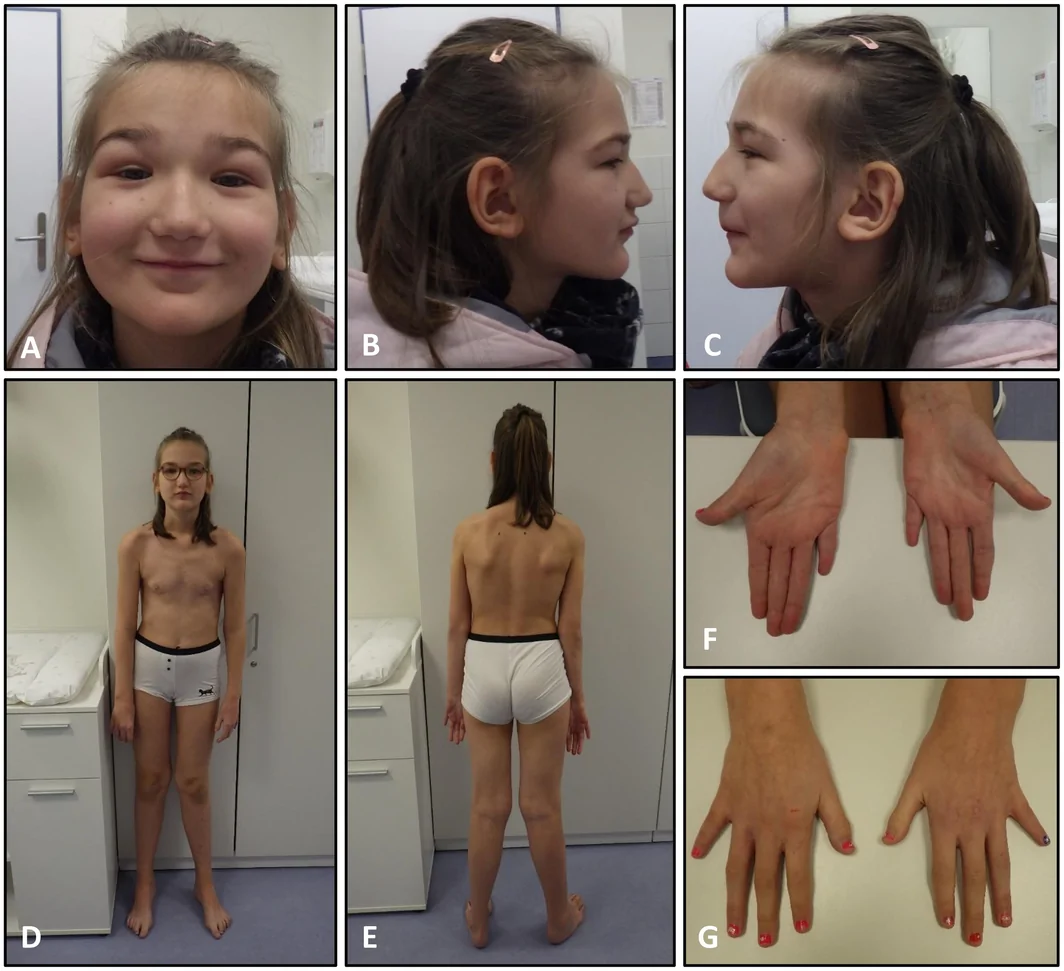
12‐year‐old female with hypertelorism, narrow palpebral fissures, large and posteriorly rotated ears (A–C), genu valgum and thin calves (D, E), slender fingers with short first and fifth metacarpals resulting in short thumb and fifth finger (F, G).

**TABLE 1:** Clinical comparison of the index patient with previously published cases with core features of *EED*‐related overgrowth syndrome (Cohen–Gibson syndrome). Each feature is indicated as present (+), absent (−), or uncertain (?).

Clinical features EED‐related overgrowth (GeneReviews)	Patient 1 (Cohen et al. 2015)	Patient 2 (Cohen and Gibson 2016)	Patient 3 (Cooney et al. 2017)	Patient 4 (Imagawa et al. 2017)	Patients 5 and 6 (Tatton‐Brown et al. 2017)	Patient 7 (Smigiel et al. 2018)	Patient 8 (Griffiths et al. 2019)	Patient 9 (Griffiths et al. 2019)	Patient 10 (Griffiths et al. 2019)	Patient 11 (Spellicy et al. 2019)	Patient 12 (Spellicy et al. 2019)	Patient 13 (Spellicy et al. 2019)	Patient 14 (Proband) (Kang and Kim 2021)	Patient 15 (younger sister) (Kang and Kim 2021)	Patient 16 (younger brother) (Kang and Kim 2021)	Patient 17 (Mother) (Kang and Kim 2021)	Patient 18 (Hetzelt et al. 2022)	Patient 19 (Proband) (Goel et al. 2024)	Patient 20 (deceased son) (Goel et al. 2024)	Patient 21 (daughter) (Goel et al. 2024)	Patient in this study	Frequency
Variant	c.906A > C; p.(Arg302Ser)	c.772C > T; p.(His258Tyr)	c.904A > G; p.(Arg302Gly)	c.707G > C; p.(Arg236Thr)	c.581A > G; p.(Asn194Ser) and c.706A > G; p.(Arg236Gly)	c.917_919delinsCGG; p.(Arg306_Asn307delinsThrAsp)	c.581A > G; p.(Asn194Ser)	c.706A > G; p.(Arg236Gly)	c.710A > G; p.(Asp237Gly)	c.773A > T; p.(His258Leu)	c.581A > G; p.(Asn194Ser)	c.772 C > T; p.(His258Tyr)	c.581A > G; p.(Asn194Ser)	c.581A > G; p.(Asn194Ser)	c.581A > G; p.(Asn194Ser)	c.581A > G; p.(Asn194Ser)	c.581A > G; p.(Asn194Ser)	c.710A > G; p.(Asn237Gly)	c.710A > G; p.(Asn237Gly)	c.710A > G; p.(Asn237Gly)	c.1091G > T; p.(Trp364Leu)	—
Suspected syndrome	Weaver syndrome	*EED*‐associated overgrowth syndrome	Weaver syndrome	Weaver syndrome	*EED*‐associated overgrowth syndrome	Weaver syndrome	*EED*‐associated overgrowth syndrome	*EED*‐associated overgrowth syndrome	*EED*‐associated overgrowth syndrome	*EED*‐associated overgrowth syndrome	*EED*‐associated overgrowth syndrome	*EED*‐associated overgrowth syndrome	Cohen–Gibson syndrome	Cohen–Gibson syndrome	Cohen–Gibson syndrome	Cohen–Gibson syndrome	Cohen–Gibson syndrome	Cohen–Gibson syndrome	Cohen–Gibson syndrome	Cohen–Gibson syndrome	Cohen–Gibson syndrome	−
Growth																						
Fetal or early childhood overgrowth/overgrowth	+	+	+	+	+	+	+	+	+	+	+	+	+	+	+	+	+	+	+	+	+	22/22
Macrocephaly	+	+	+	+	?	+	+	+	+	+	−	+	−	?	?	?	+	−	+	−	−	12/17
Large hands and feet	+	+	+	+	?	+	?	+	?	+	+	+	+	?	?	?	+	+	+	+	+	15/15
Advanced bone age	+	+	+	+	?	+	?	?	?	+	−	+	−	?	?	?	?	?	?	?	−	7/10
Development																						
Global developmental delay and ID	+	+	+	+	+	+	+	+	+	+	+	+	+	+	+	+	+	+	+	+	+	22/22
Gross motor delay	+	+	+	?	?	+	?	?	?	+	?	?	?	?	?	?	?	?	?	?	+	6/6
Fine motor delay	+	+	+	?	?	?	?	?	?	−	−	+	?	?	?	?	?	?	?	?	+	5/7
Language disorder	+	+	+	?	?	+	?	+	?	−	−	+	?	?	?	?	+	+	+	+	+	11/13
Social development delay	+	+	+	?	?	?	?	?	?	?	?	?	?	?	?	?	−	?	?	?	+	4/5
Skeletal (radiologic)																						
Advanced osseous maturation	+	+	+	+	?	+	?	?	?	+	−	−	−	?	?	?	?	?	?	?	−	6/10
Metaphyseal flaring/widening/radiolucency	+	?	+	+	?	?	?	?	?	−	+	?	?	?	?	?	?	?	?	?	−	4/6
Cervical spine abnormalities	−	+	+	?	?	?	−	+	−	+	?	?	?	?	?	?	?	?	?	?	−	4/8
Flattened metaphyses of long bones	+	?	?	?	?	?	?	?	?	?	?	?	?	?	?	?	?	?	?	?	−	1/2
Coxa valga/hip dislocation/leg length discrepancy	−	?	+	+	?	?	?	?	?	−	−	?	?	?	?	?	?	?	?	?	+	3/6
Osteopenia	+	+	+	?	?	?	?	?	?	−	−	?	?	?	?	?	?	?	?	?	−	3/6
Joint hypermobility	?	+	+	?	?	+	?	?	?	+	−	+	−	?	?	?	+	?	?	?	−	6/9
Craniofacial																						
Round face/broad forehead	+	+	+	+	?	+	+	+	+	+	−	+	+	+	+	+	+	+	+	+	+	19/20
Hypertelorism	+	+	+	+	−	+	−	+	−	+	+	−	+	+	+	+	+	+	+	+	+	17/22
Depressed/low/wide nasal bridge	+	−	+	+	?	+	+	+	−	+	+	−	+	+	+	+	?	−	−	−	+	13/19
Large/prominent ears	+	+	+	+	?	+	+	+	?	+	+	+	?	?	?	?	+	+	+	+	+	15/15
Retrognathia/micrognathia	+	+	+	+	+	+	+	−	−	−	−	−	?	?	?	?	+	−	?	+	+	11/17
Prominent philtrum/horizontal chin crease	+	−	+	+	?	+	+	+	+	+	−	−	+	+	+	+	+	+	+	+	−	16/20
Facial hypotonia/open mouth appearance	+	−	+	+	?	+	+	−	−	−	−	−	?	?	?	?	?	?	?	?	−	5/12
Epicanthus	−	−	+	+	?	+	+	−	−	?	−	−	?	?	?	?	?	?	?	−	+	5/12
High anterior hairline	−	+	−	−	?	+	−	−	+	−	+	+	?	?	?	?	?	−	−	+	−	6/15
Ophthalmologic																						
Ophthalmological abnormalities	+	?	+	?	?	+	+	+	+	+	+	+	+	?	?	?	+	+	+	?	+	14/14
Myopia	+	−	−	?	?	?	?	?	?	+	+	−	?	?	?	?	+	+	+	?	+	7/10
Strabismus	−	−	+	?	?	?	?	?	?	+	−	−	+	?	?	?	?	?	+	?	−	4/9
Ptosis	−	−	+	?	?	?	+	+	+	−	+	+	?	?	?	?	?	?	?	?	−	6/10
Other systemic																						
Scoliosis/kyphosis	+	+	−	−	?	+	−	−	+	+	+	?	?	?	?	?	+	?	?	?	+	8/12
Hernias	+	+	+	+	?	−	+	?	?	−	−	+	?	?	?	?	?	?	?	?	−	6/10
Feeding difficulties	−	−	+	?	?	?	?	?	?	?	?	?	?	?	?	?	?	?	?	?	−	1/4
Hypotonia (central and/or peripheral)	+	+	+	−	?	+	?	+	?	−	−	?	?	?	?	?	?	+	+	+	+	9/12
Cardiovascular																						
Congenital heart defect (ASD/VSD/PDA)	?	−	+	+	?	?	−	+	−	+	+	−	−	+	+	−	+	?	?	?	?	8/14
Valvular anomalies/murmur	+	−	?	?	?	?	?	?	?	?	?	?	−	?	?	?	?	?	?	?	+	2/4
Endocrine/other																						
Normal endocrine labs	+	+	?	−	?	?	−	?	?	+	+	+	+	?	?	?	?	?	?	?	+	7/9
Epilepsy	+	−	−	−	−	−	+	−	−	−	−	−	+	−	−	+	+	+	+	+	−	8/22

Endocrinological evaluation for tall stature, including laboratory assessments, yielded unremarkable results. Chromosomal analysis revealed a normal female karyotype (46,XX). Clinical suspicion was raised for an overgrowth–intellectual disability syndrome. A definitive molecular diagnosis was not established at the time of the initial assessment.

To further support the molecular diagnosis, the patient's facial gestalt was analyzed using two AI‐based tools: GestaltMatcher and Face2Gene (Hsieh et al. 2022; Lesmann et al. 2024). In GestaltMatcher (Model version v1.1.0, Gallery version 20.08.2024), Borjeson–Forssman–Lehmann syndrome was listed as the top suggested disorder, while Cohen–Gibson syndrome (COGIS) ranked second in similarity (Figure S1). This finding is of particular relevance, as it aligns with the suspected clinical diagnosis. Additionally, analysis via Face2Gene placed Cohen–Gibson syndrome under the “Ultra‐Rare” category, again highlighting it with medium Gestalt similarity (76%) (Figure S2).

Radiographic evaluation at 10 years and 4 months of age (height at the time: approximately 166 cm) demonstrated a skeletal age of 10 years, consistent with chronological age. Based on the Bayley and Pinneau method, the projected adult height was estimated at 192 cm, significantly exceeding the mid‐parental target height of 163 cm, calculated using the parental heights of 164 cm (mother, ~75th percentile; +0.67 SD) and 175 cm (father, ~50th percentile; 0 SD). The patient's 16‐year‐old healthy brother measured 183 cm (approximately 90th percentile; +1.28 SD).

### 3.2 Genetic Analysis

Whole exome sequencing (WES) was performed on DNA extracted from peripheral blood of the patient. A likely pathogenic variant was identified in the *EED* gene (NM_003797.5), located in exon 10, resulting in a missense variant: c.1091G > T; p.(Trp364Leu). The variant was detected at a variant allele fraction (VAF) of approximately 28% in the blood‐derived DNA of the patient (Table 2). It was not detected in either parental sample, confirming its de novo origin. The very low‐level variant reads observed in the parental samples (17/26748 and 25/30937, representing mutant/total reads) are consistent with sequencing artefacts. The de novo status was supported by high sequencing depth and a validated detection threshold of approximately 1%, providing no evidence of parental mosaicism in blood above this threshold. Visualization of the sequencing data in the Integrative Genomics Viewer (IGV) is shown in Figure 2.

**TABLE 2:** Detection of the *EED* variant in different tissues from the index patient and her parents using whole‐exome sequencing and amplicon‐based deep sequencing (Nextera).

Method	Blood (VAF, mutant/total reads)	Buccal mucosa swab 1 (VAF, mutant/total reads)	Buccal mucosa swab 2 (VAF, mutant/total reads)	Saliva (VAF, mutant/total reads)
Index patient				
Exome	28% (64/232)	n.a.	n.a.	n.a.
Amplicon‐based deep sequencing (Nextera)	31% (9.013/29,261)	11% (2,504/21,828)	17% (3,035/18,069)	30% (6,710/22,733)
Father				
Amplicon‐based deep sequencing (Nextera)	0% (17/26,748)	n.a.	n.a.	n.a.
Mother				
Amplicon‐based deep sequencing (Nextera)	0% (25/30,937)	n.a.	n.a.	n.a.

Abbreviations: n.a. = not analyzed; VAF = variant allele fraction (mutant reads/total reads).

**FIGURE 2:**
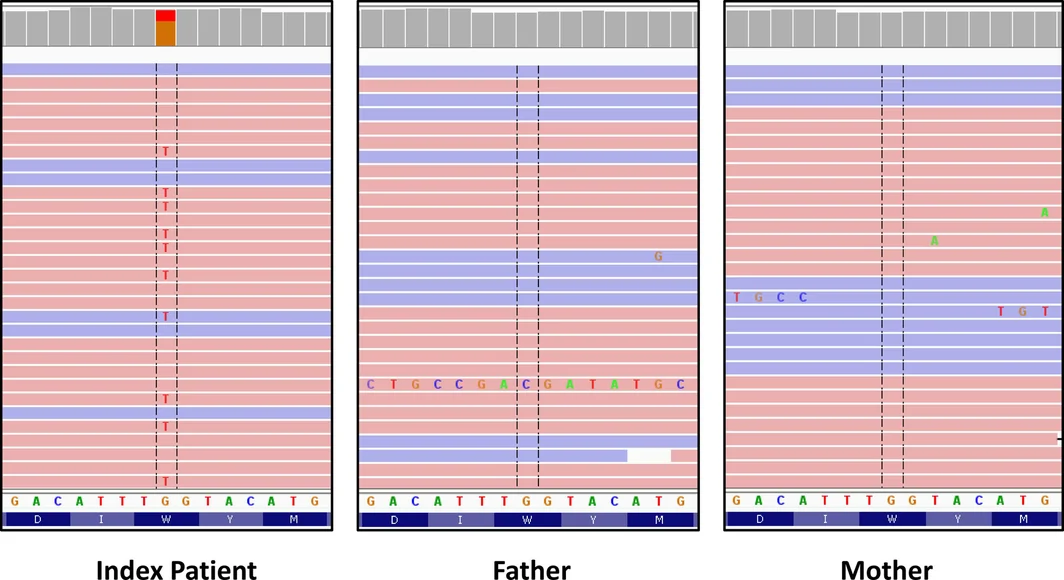
IGV screenshot of amplicon‐based deep sequencing (Nextera) of the *EED* gene (NM_003797.5) in the index patient, father, and mother. The index patient carries a missense variant c.1091G > T; p.(Trp364Leu) with a variant allele fraction of 31% (total read count: 29,261). This variant is absent in the sequencing data of both the father and the mother (total read counts: 26,748 and 30,937, respectively). Sequencing reads are displayed as horizontal bars, with reference bases indicated by color‐coded nucleotides.

Given the relatively low VAF observed in the initial analysis, which was suggestive of somatic mosaicism, confirmatory testing was performed using Nextera‐based targeted sequencing to achieve higher coverage at the locus of interest. This analysis confirmed the presence of the same *EED* variant with a VAF of 31% in blood‐derived DNA, supporting the initial WES finding and strengthening the hypothesis of somatic mosaicism (Table 2).

To further investigate the distribution of the variant across different tissues, DNA was extracted from two independently collected buccal mucosa samples, one obtained using a dry sterile swab and the other using an Oracollect•DNA OCR‐100 swab, as well as from one saliva sample. The variant was detected in the buccal samples at a VAF of 11% and 17%, respectively, and in the saliva sample at a frequency of 30% (Table 2). These findings are consistent with a postzygotic mutational event and support the diagnosis of mosaic *EED*‐related overgrowth syndrome.

The variant c.1091G>T; p.(Trp364Leu) identified in *EED* is extremely rare in population databases, being found only once in gnomAD v4.1.0 (1/1,591,280 alleles; allele frequency: 6.284 × 10^−7^), and has not been reported in ClinVar or HGMD Professional 2023.4. Inspection of gnomAD IGV data suggests mosaicism with a VAF of approximately 25%. In silico tools, including AlphaMissense (score: 0.9997), CADD (score: 29.8), PolyPhen (max) (score: 1), REVEL (score: 0.538), PhyloP (score: 8.79), PrimateAI‐3D (score: 0.87; gene‐specific threshold: 0.42), and SIFT (max) (score: 0), predicted a predominantly deleterious effect. The amino acid change occurs within the WD40 repeat domain and affects a conserved residue (Trp364) that forms part of the aromatic cage responsible for binding trimethylated H3K27; this interaction is crucial for the allosteric activation and function of the PRC2 complex and thus may be relevant to its stability and activity (Ju et al. 2023; Xu et al. 2010). No other pathogenic or likely pathogenic variants relevant to the patient's phenotype were identified in the exome data.

According to ACMG/AMP guidelines, the variant was classified as likely pathogenic, based on the following criteria: PS2 (confirmed de novo), PM2_Supporting (absent or extremely rare in controls), PP3 (multiple lines of computational evidence supporting a deleterious effect), and PP4 (phenotype highly specific for disease known to be caused by (likely) pathogenic variants in this gene).

### 3.3 Pathogenic Variant Landscape and Literature Review

The first germline pathogenic variant in the *EED* gene associated with an overgrowth phenotype was reported in 2015, and only a small number of individuals have been described since, highlighting the ultra‐rare nature of *EED*‐related neurodevelopmental disorders (Cohen et al. 2015). To the best of our knowledge, to date, 11 disease‐causing variants in the *EED* gene associated with overgrowth syndromes and neurodevelopmental disorders have been reported in the literature (Figure 3). They are distributed across several functionally important residues, with recurrent pathogenic variants suggesting potential mutational hotspots. The majority of variants are missense variants affecting conserved residues within the EED protein, although one small indel has also been reported (Figure 3). Notably, all reported variants preserve the reading frame of *EED*, likely resulting in the production of altered EED proteins, as no frameshift or nonsense variants have been identified to date. The variants described so far are presented in Table 1.

**FIGURE 3:**
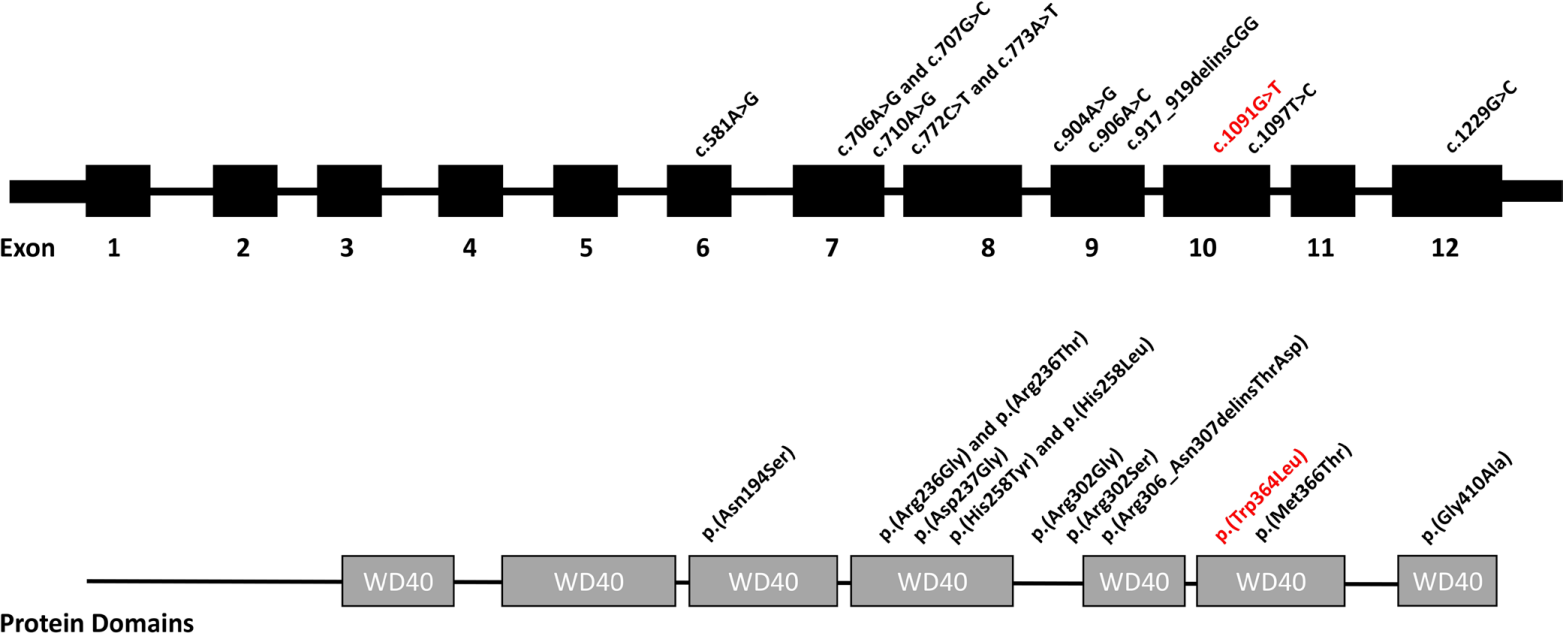
Schematic representation of the *EED* gene structure (NM_003797.5), illustrating the positions of reported variants across exons 1–12 and the corresponding protein domains. Coding exons are depicted as black boxes; the first and last exons include the 5′ and 3′ untranslated regions (UTRs), respectively. All identified variants are labeled with their respective cDNA positions; the mosaic variant identified in this study is highlighted in red. The lower part of the figure shows the structure of the encoded protein, with WD40 domains represented as grey boxes.

### 3.4 Episignature

Genome‐wide DNA methylation analysis using the EpiSign METRIC platform revealed a methylation profile consistent with the episignature of PRC2 complex disorders that include the *EED*‐associated syndrome and EZH2‐associated Weaver syndrome, with high confidence. In the hierarchical clustering heatmap, the patient sample showed a methylation pattern concordant with the PRC2 reference cohort and distinct from signature controls (Figure 4A). In the multidimensional scaling (MDS) plot, the patient sample clustered closely with the PRC2 reference cohort and was clearly separated from signature controls (Figure 4B). A comparable pattern was observed in the t‐distributed stochastic neighbor embedding (t‐SNE) plot, in which the patient sample again localized within the PRC2 cluster rather than with controls or batch samples (Figure 4C). Overall, the observed methylation profile provided functional support for the likely pathogenic classification of the identified *EED* variant.

**FIGURE 4:**
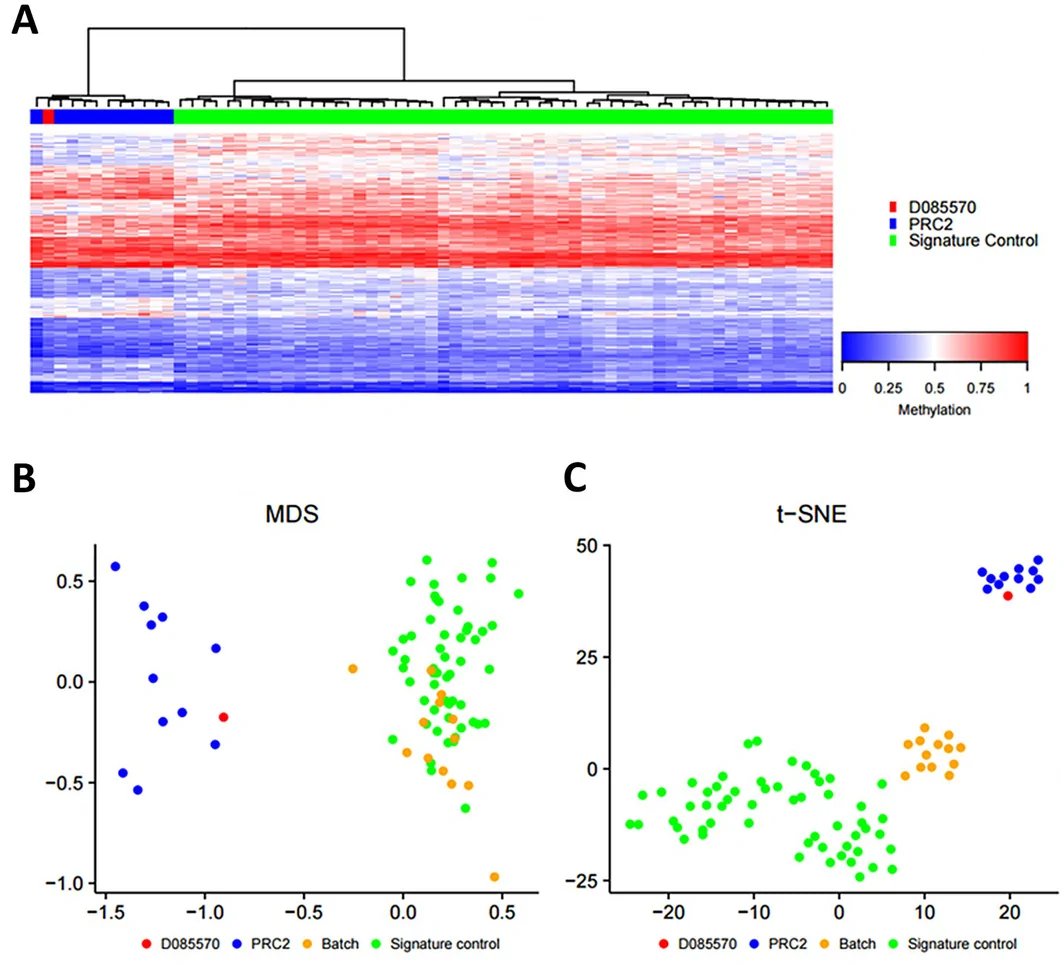
EpiSign METRIC results for the index patient. (A) Hierarchical clustering heatmap of differentially methylated sites comparing the patient sample (red) with the PRC2 reference cohort (blue) and signature controls (green). The dendrogram indicates clustering based on similarity of genome‐wide methylation profiles. The patient sample clusters with the PRC2 reference cohort and is clearly separated from signature controls. Methylation values are displayed on a scale from hypomethylated (blue) to hypermethylated (red). (B) Multidimensional scaling (MDS) plot showing clustering of the patient sample (red) with the PRC2 reference cohort (blue), clearly separated from signature controls (green). Batch samples are shown in orange. (C) t‐distributed stochastic neighbor embedding (t‐SNE) plot demonstrating a comparable clustering pattern, with the patient sample localizing within the PRC2 cluster and apart from both signature controls and batch samples.

## 4 Discussion

In this study, we report an affected individual with an *EED*‐related overgrowth–intellectual disability syndrome caused by postzygotic somatic mosaicism. The likely pathogenic variant was identified by exome sequencing and validated through deep targeted sequencing across multiple tissue types, with VAFs of 31% in blood, 30% in saliva, and 11%–17% in buccal mucosa. These findings highlight the diagnostic value of multi‐tissue analysis in detecting mosaic variants, as reliance on blood alone may underestimate the true mutational burden. The relatively high VAFs across tissues of both mesodermal and ectodermal origin support the likely pathogenic relevance of the variant and suggest an early embryonic mutational event.

Phenotypically, the patient exhibits a spectrum of features characteristic of PRC2 component dysfunction, including prenatal and postnatal overgrowth, distinctive craniofacial features, congenital anomalies, and developmental delay. While overlapping with Weaver syndrome, certain dysmorphic findings, such as epicanthus inversus, narrow palpebral fissures with ptosis, micrognathia, and prominent ears, are more consistent with *EED*‐related disorders, especially Cohen–Gibson syndrome. Our patient presented with additional anomalies such as cardiac anomalies, musculoskeletal abnormalities, hypotonia, and coordination issues, reflecting the systemic effects of PRC2 dysregulation as reported in previous cases involving pathogenic *EED* variants (Cohen and Gibson 2016; Cohen et al. 2015; Goel et al. 2024).

Molecularly, the variant's presence across multiple tissues supports a mosaic distribution and aligns with a postzygotic origin. The clinical presentation, including the results from GestaltMatcher, the de novo status, and computational predictions collectively provide strong evidence supporting the likely pathogenic classification of the variant. The variant is present once in gnomAD v4.1.0, indicating that it is extremely rare. The presence of a PRC2 complex‐associated episignature, as revealed by DNA methylation analysis, further corroborates the likely pathogenic classification of the identified variant.

AI‐based facial analysis (GestaltMatcher and Face2Gene) supported the molecular findings and contributed to prioritizing the candidate gene during variant analysis. This clinically guided evaluation, together with sufficient sequencing depth and a relatively high VAF (~30%) in blood, likely facilitated the detection of the mosaic variant.

However, several limitations should be acknowledged. This report reflects a single‐case observation, and no functional assays were performed to directly demonstrate the cellular consequences of the *EED* variant. Moreover, the extent to which the degree of mosaicism correlates with phenotypic severity remains unresolved. In our patient, mosaic VAFs ranged from 11% to 31% across different tissues, and the phenotype was of moderate severity with multisystemic involvement, suggesting that even mosaicism at intermediate levels may result in clinically relevant manifestations. These gaps highlight the need for systematic studies of mosaicism in PRC2‐related disorders using comprehensive molecular and clinical phenotyping.

In summary, we advocate for the routine consideration of mosaicism in the differential diagnosis of PRC2‐related syndromes, particularly in patients with unexplained or atypical features. Future research should focus on systematically investigating mosaicism in larger cohorts and developing standardized approaches to assess its clinical relevance. Deeper insights into genotype–phenotype relationships in mosaic cases will ultimately enhance diagnostic accuracy and inform patient care.

## Author Contributions

F.S. and M.M. curated the data, performed data analysis and interpretation, and drafted the manuscript. F.S., M.M., B.B., W.V., T.N., L.S., and D.W. contributed to interpreting the results and revising the manuscript. D.W. conceived the study, contributed to the interpretation of the results, and provided overall supervision. All authors read and approved the final version of the manuscript.

## Funding

This work was supported by the Deutsche Forschungsgemeinschaft (DFG, German Research Foundation), WI 1440/8‐1, RE 4149/1‐1.

## Consent

Informed consent was obtained from the patient and her parents for the publication of clinical details and images.

## Conflicts of Interest

The authors declare no conflicts of interest.

## Supporting information


**Figure S1:** Output from GestaltMatcher (v1.1.0) showing Cohen–Gibson Syndrome ranked second, with BFLS and SBBYSS among the top suggested syndromes.

**Figure S2:** Output from Face2Gene showing Cohen–Gibson Syndrome listed under the “Ultra‐Rare” category with 76% Gestalt similarity.

## Data Availability

The data supporting the findings of this study are available from the corresponding author upon reasonable request.
